# Quantitative Visualization of Gene Expression in Mucoid and Nonmucoid Pseudomonas aeruginosa Aggregates Reveals Localized Peak Expression of Alginate in the Hypoxic Zone

**DOI:** 10.1128/mBio.02622-19

**Published:** 2019-12-17

**Authors:** Peter Jorth, Melanie A. Spero, J. Livingston, Dianne K. Newman

**Affiliations:** a Division of Biology and Biological Engineering, California Institute of Technology, Pasadena, California, USA; b Division of Geological and Planetary Sciences, California Institute of Technology, Pasadena, California, USA; University of Washington

**Keywords:** HCR, *Pseudomonas aeruginosa*, aggregate, biofilms, gene expression, *in situ* hybridization, microscopy

## Abstract

It is well appreciated that oxygen- and other nutrient-limiting gradients characterize microenvironments within chronic infections that foster bacterial tolerance to treatment and the immune response. However, determining how bacteria respond to these microenvironments has been limited by a lack of tools to study bacterial functions at the relevant spatial scales *in situ*. Here, we report the application of the hybridization chain reaction (HCR) v3.0 to provide analog mRNA relative quantitation of Pseudomonas aeruginosa single cells as a step toward this end. To assess the potential for this method to be applied to bacterial populations, we visualized the expression of genes needed for the production of alginate (*algD*) and the dissimilatory nitrate reductase (*narG*) at single-cell resolution within laboratory-grown aggregates. After validating new HCR probes, we quantified *algD* and *narG* expression across microenvironmental gradients within both single aggregates and aggregate populations using the agar block biofilm assay (ABBA). For mucoid and nonmucoid ABBA populations, *narG* was expressed in hypoxic and anoxic regions, while alginate expression was restricted to the hypoxic zone (∼40 to 200 μM O_2_). Within individual aggregates, surface-adjacent cells expressed alginate genes at higher levels than interior cells, revealing that alginate expression is not constitutive in mucoid P. aeruginosa but instead varies with oxygen availability. These results establish HCR v3.0 as a versatile and robust tool to resolve subtle differences in gene expression at spatial scales relevant to microbial assemblages. This advance has the potential to enable quantitative studies of microbial gene expression in diverse contexts, including pathogen activities during infections.

## Abstract (precis)

A goal for microbial ecophysiological research is to reveal microbial activities in natural environments, including sediments, soils, or infected human tissues. Here, we report the application of the hybridization chain reaction (HCR) v3.0 to quantitatively measure microbial gene expression *in situ* at single-cell resolution in bacterial aggregates. Using quantitative image analysis of thousands of Pseudomonas aeruginosa cells, we validated new P. aeruginosa HCR probes. Within *in vitro* P. aeruginosa aggregates, we found that bacteria just below the aggregate surface are the primary cells expressing genes that protect the population against antibiotics and the immune system. This observation suggests that therapies targeting bacteria growing with small amounts of oxygen may be most effective against these hard-to-treat infections. More generally, this proof-of-concept study demonstrates that HCR v3.0 has the potential to identify microbial activities *in situ* at small spatial scales in diverse contexts.

## OBSERVATION

Despite decades of research that has elucidated mechanisms of bacterial virulence, antibiotic tolerance, and antibiotic resistance, many infections remain impossible to eradicate. Phenotypic heterogeneity likely plays an important role in the failure of drugs and the immune system to clear chronic infections. Chronic Pseudomonas aeruginosa lung infections in people with cystic fibrosis (CF) are a prime example. Within individual lobes of the CF lung, genetically antibiotic-susceptible and -resistant P. aeruginosa sibling bacteria coexist (1). This likely affects treatment, because resistant bacteria can protect susceptible bacteria when mixed together *in vitro* (2, 3). Likewise, CF lung mucus contains steep oxygen gradients, and anoxic conditions reduce antibiotic susceptibility (4–7). While we know that bacterial genetic diversity and infection site chemical heterogeneity exist, tools to measure bacterial phenotypes *in situ* are lacking. Here, we applied the third generation of the hybridization chain reaction (HCR v3.0) to quantitatively measure gene expression in P. aeruginosa in an *in vitro* aggregate model system. Our findings suggest that HCR v3.0 could prove to be a useful tool for analyzing *in situ* bacterial gene expression in virtually any species in any context.

## 

### Validation of HCR v3.0 probes for quantifying P. aeruginosa gene expression.

**FIG 1:**
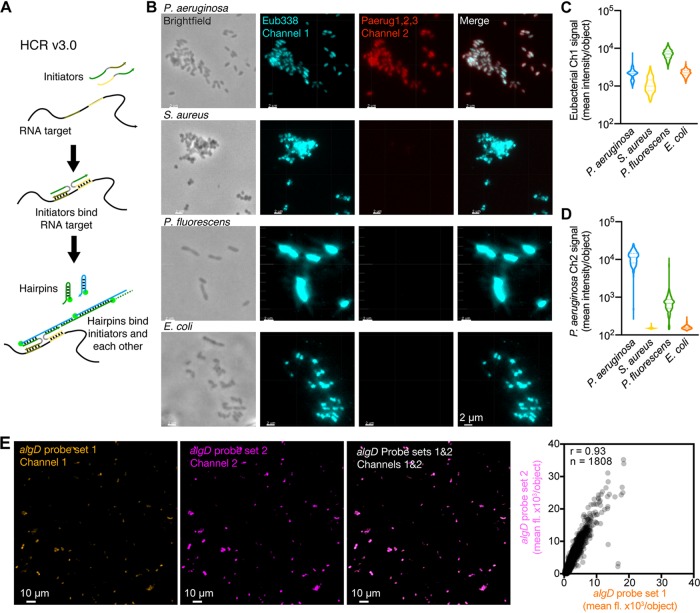
HCR v3.0 analysis is specific and quantitative. (A) HCR v3.0 utilizes probe pairs that address proximal subsequences of the target RNA. Each probe within a pair carries one-half of an HCR initiator so that cognate binding to the target colocalizes a full HCR initiator. Fluorophore-labeled HCR amplification hairpins are kinetically trapped so that they do not polymerize until they encounter a full HCR initiator, generating a fluorescent amplification polymer tethered to a specifically bound probe pair. As a result, using HCR v3.0 reagents, individual probes and hairpins that bind nonspecifically in the sample do not generate amplified background. (B) High selectivity using HCR v3.0 to detect rRNAs across bacterial species. Micrographs show that the Eub338 probe pair (turquoise) binds P. aeruginosa, S. aureus, P. fluorescens, and E. coli rRNA as intended, while the P. aeruginosa Paerug probe set (red) selectively binds P. aeruginosa rRNA. Scale bars, 2 μm. (C) Quantification of single-cell Eub338 fluorescence intensities shows the intended broad selectivity (violin plots summarize data from 3 micrographs per organism). (D) Quantification of single-cell Paerug fluorescence intensities shows that Paerug probes are selective to *Pseudomonas* spp., with ∼10-fold higher signal intensity produced by P. aeruginosa than by P. fluorescens and even higher selectivity against the more distantly related E. coli and S. aureus (violin plots summarize data from 3 micrographs per organism). (E) Representative two-channel single-cell HCR analysis using two *algD* probe sets. Highly correlated signal (*r* = 0.93, Pearson correlation, *n* = 1,808 cells analyzed); linear distribution and small intercept characterize accuracy, and scatter around the line characterizes precision (9, 12). Ten probe pairs were used for each *algD* probe set to target *algD* mRNA in the PAO1 Δ*algD*/pMQ72::*algD* strain after *algD* expression induction with 0.10% l-arabinose. Scale, 10 μm. Graph is representative of five analyses of replicates using 0.10% l-arabinose to induce *algD* expression; median Pearson correlation, *r* = 0.93 (see Fig. S3 in the supplemental material for other replicates). See also Fig. S1, S2, and S3.

As a first step, we tested HCR probe specificity and quantitation. HCR is a fluorescent *in situ* hybridization method that includes a quantitative, enzyme-free signal amplification step to help visualize low-abundant RNAs (8, 9). We previously used single HCR v2.0 probes to detect bacterial taxa in CF sputum samples (10), and HCR v2.0 was also used by Nikolakakis et al. to detect host and bacterial mRNAs in the Hawaiian bobtail squid-Vibrio fischeri symbiosis (11). We chose to apply HCR v3.0 as a tool to quantify bacterial gene expression *in situ* because of its improved selectivity over HCR v2.0. HCR v3.0 requires pairs of probes to hybridize to adjacent binding sites on the target RNA in order to colocalize a full HCR initiator and trigger growth of an HCR amplification polymer (Fig. 1A), ensuring that individual probes will not generate amplified background even if they bind nonspecifically in the sample (8, 9). We designed and validated HCR v3.0 probe sets that could be used to (i) differentiate species by targeting rRNAs and (ii) measure gene expression by targeting mRNAs.

10.1128/mBio.02622-19.1 FIG S1 Individual P. aeruginosa HCR v3.0 probes are specific. (A) Fluorescence micrographs of P. aeruginosa FRD875 probed with Eub338 (green) and either Paerug1, Paerug2, or Paerug3 (magenta). (B) Fluorescence micrographs of S. aureus MN8 probed with Eub338 (green) and either Paerug1, Paerug2, or Paerug3 (magenta). Individual Paerug1, Paerug2, and Paerug3 probe pairs bound to P. aeruginosa but not S. aureus. Scale bars, 1 μm. Download FIG S1, TIF file, 2.6 MB. Copyright © 2019 Jorth et al. 2019 Jorth et al. This content is distributed under the terms of the Creative Commons Attribution 4.0 International license.

10.1128/mBio.02622-19.2 FIG S2 Initiators probes do not induce amplification when only one probe in the initiator pair is used. HCR v3.0 initiator probes come in pairs of odd and even initiators. P. aeruginosa FRD1 was probed with just the odd initiator probe from the Eub338 HCR v3.0 probe pair with all of the odd initiator probes from the 20 pair *algD* probe set (top images) and with the odd initiator probe from the Eub338 HCR v3.0 probe pair with the 3 odd initiator probes from the Paerug1 to -3 probe set (bottom images). None of the odd initiator probes individually, or in groups, were capable of inducing fluorescence amplification with the fluorescent hairpin probes when used without the even probe pairs. Download FIG S2, TIF file, 1.7 MB. Copyright © 2019 Jorth et al. 2019 Jorth et al. This content is distributed under the terms of the Creative Commons Attribution 4.0 International license.

10.1128/mBio.02622-19.3 FIG S3 Two-channel *algD* HCR analysis verifies that new P. aeruginosa *algD* HCR probes are quantitative. Single-cell fluorescent signals for the PAO1 Δ*algD*/pMQ72::*algD* complement strain with 0.10% l-arabinose inducer for *algD* probe set 1 (orange) and *algD* probe set 2 (magenta). Pearson correlations (*r*) and numbers of cells analyzed (*n*) for each micrograph are indicated. Pearson correlation coefficient for the 5th replicate increases from *r* = 0.84 to *r* = 0.95 if the single outlier in the lower right is excluded. Each scatter plot represents data from a unique micrograph after *algD* expression was induced with 0.10% l-arabinose. Download FIG S3, TIF file, 1.2 MB. Copyright © 2019 Jorth et al. 2019 Jorth et al. This content is distributed under the terms of the Creative Commons Attribution 4.0 International license.

To test specificity, we designed one HCR v3.0 probe set to detect 16S rRNA in all eubacteria (1 probe pair) and another HCR v3.0 probe set (3 probe pairs) to detect P. aeruginosa specifically, using our previous HCR v2.0 probe sets as starting points (10). P. aeruginosa, Pseudomonas fluorescens, Escherichia coli, and Staphylococcus aureus were grown *in vitro* in LB broth, fixed with paraformaldehyde, incubated with the eubacterial and P. aeruginosa*-*specific probes, washed to remove excess probes, incubated with fluorescent HCR amplifiers, washed to remove excess HCR amplifiers, and imaged via confocal microscopy. Single-cell fluorescence intensities were quantified computationally with Imaris (see methods in the supplemental material). When only one probe from each probe pair was used, no fluorescence was observed, demonstrating the background suppression feature of HCR v3.0 probes (see Fig. S2). As expected, the eubacterial probe set detected all four organisms (Fig. 1B and C and S1) while the P. aeruginosa probe set produced a visible signal only for P. aeruginosa. Quantifying single-cell fluorescence for the P. aeruginosa probe set, intensities were an order of magnitude lower for P. fluorescens (the most closely related of the three off-target strains) and 2 orders of magnitude lower for Escherichia coli and Staphylococcus aureus (Fig. 1D), demonstrating high selectivity for the intended bacteria.

Next, we tested the utility of HCR v3.0 for performing mRNA relative quantitation for single P. aeruginosa cells within a population. According to Trivedi et al. (12), we performed a 2-channel redundant detection experiment in which the *algD* mRNA was simultaneously detected using two probe sets that trigger different HCR amplifiers carrying spectrally distinct fluorophores (each probe set comprised 10 HCR v3.0 probe pairs that bind to different subsequences along the mRNA). Because the HCR signal scales approximately linearly with the number of target mRNAs per cell (12), we expected a 2-channel scatter plot of single-cell fluorescent signal to yield an approximately linear distribution. In this setting, accuracy corresponds to linearity with zero intercept and precision corresponds to the scatter around the line (12). We generated a range of *algD* single-cell expression levels by overexpressing *algD* from the arabinose-inducible expression plasmid pMQ72 in a P. aeruginosa Δ*algD* mutant (13, 14) and analyzed the 2-channel scatter plot for more than ∼8,000 cells in confocal micrographs. As expected, the single-cell signals were highly correlated between the two channels (median Pearson *r* = 0.93 for *N* = 5 micrographs) (Fig. 1E and S3), and the distribution was approximately linear with a small intercept and tight scatter around the line. These results are consistent with previous validation studies demonstrating that HCR v3.0 enables accurate and precise relative mRNA quantitation in cells and embryos (8, 9). Note that the precision increases with probe set size (12), and so it is beneficial to maximize the probe set size to the extent permitted by selectivity requirements imposed by the transcriptomes present in the study.

### HCR reveals alginate gene expression in hypoxic zones of P. aeruginosa aggregates.

As a case study, we chose to measure P. aeruginosa alginate (*algD*) and nitrate reductase (*narG*) gene expression in aggregates formed by mucoid (FRD1) and nonmucoid (PA14) strains. This approach was chosen for several reasons. First, measuring *algD* expression *in situ* is of interest because alginate is overproduced by mucoid strains in CF lung infections (15, 16), and mucoid strains are associated with worsened lung function (17). Second, as a technical control, the *algD* gene should be more highly expressed in the mucoid than in the nonmucoid strain and produce a stronger HCR signal (15). Third, previous research suggests that alginate may be expressed under hypoxic and anoxic conditions in mucoid isolates (7, 18–20), yet the precise location of alginate gene expression in both mucoid and nonmucoid aggregate biofilms was unknown and alginate expression had not been studied at the single-cell level. We could also quantify *algD* expression relative to *narG*, a gene induced under hypoxic and anoxic conditions (18, 21), a useful reference point for mapping *algD* expression in aggregates relative to oxygen availability.

**FIG 2:**
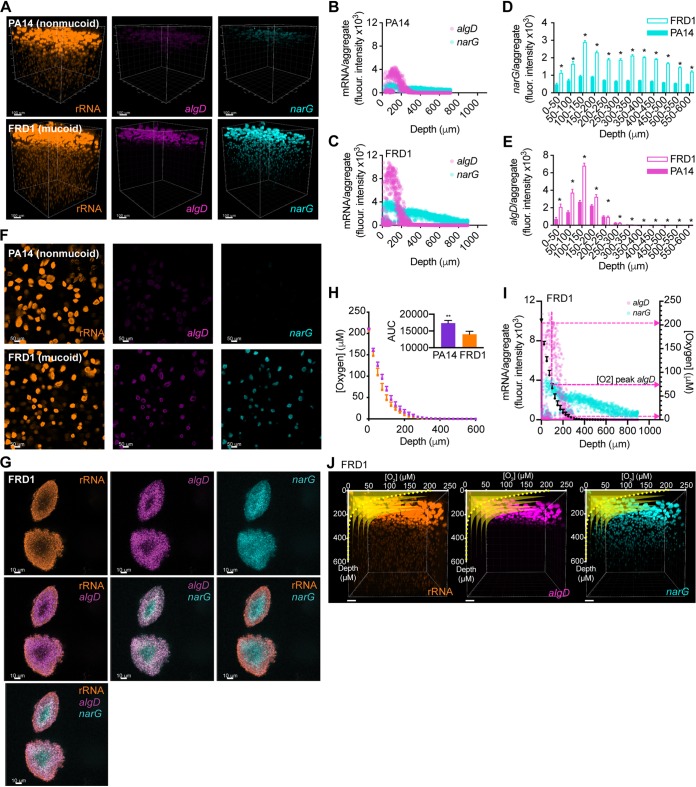
Alginate gene expression is highest in hypoxic regions of P. aeruginosa aggregates. (A) Three dimensional (3D) fluorescence micrographs of nonmucoid PA14 and mucoid FRD1 ABBA samples probed with the Eub338 (rRNA), *algD*, and *narG* HCR v3.0 probes. Scale bars, 100 μm. Mean *algD* and *narG* HCR signals per individual aggregate in nonmucoid (B) and mucoid (C) strains. Mean *narG* (D) and *algD* (E) HCR signals per ABBA aggregate biofilm at different binned depths below the air-agar interface in each sample (50-μm bins; means ± standard errors of the means [SEMs]; *P < 0.05, unpaired two-tailed *t* test). (F) Two-dimensional (2D) micrographs of nonmucoid and mucoid ABBA samples probed with the rRNA, *algD*, and *narG* HCR probes. Images correspond to single Z-slices 99 μm below the air-agar interface. Scale bars, 50 μm. (G) 2D micrographs of mucoid ABBA aggregates probed with the rRNA, *algD*, and *narG* HCR probes. Overlays show that *narG* is expressed by interior bacterial cells, while *algD* is expressed by bacterial cells just below the aggregate surface. Each image corresponds to the same Z-slice with different probes shown. Scale bars, 10 μm. (H) Oxygen profiles in nonmucoid and mucoid ABBA samples. Mean oxygen concentrations at 25-μm intervals from the air-agar interface to 600 μm below (*n* = 3) are indicated. Inset bar graph indicates area under the curve (AUC) for each scatter plot (**P < 0.005, unpaired two-tailed *t* test). (I) Mean *algD* and *narG* expression per mucoid ABBA aggregate (left *y* axis) plotted with mean oxygen concentrations measured (right *y* axis). Middle pink arrow indicates oxygen concentration at which peak *algD* expression was detected, bottom and top pink arrows indicate minimum and maximum oxygen concentrations at which *algD* expression was detected. Error bars in panels H and I indicate SEMs for the oxygen concentrations. (J) Expression of *algD* is restricted to hypoxic regions, while *narG* is detected in hypoxic and anoxic regions. Oxygen profiles (yellow) overlay 3D micrographs showing rRNA, *algD*, and *narG* HCR signals in mucoid ABBA samples. This shows *algD* gene expression is only detected in regions where oxygen is also detected, while *narG* and rRNA are detected in regions where oxygen is not measurable. Oxygen profiles are plotted multiple times using perspective at different *xz* planes along the *y* axis. In panels A to G, I, and J, data are shown from a representative ABBA experiment. In all micrographs, the power for each individual laser used to excite the different fluorophores and the gain for each detector were kept consistent from one experimental replicate to another. Thus, gene expression for individual genes (e.g., *algD* in FRD1 or PA14 strains) can be compared from one experiment to another as well as across space within individual experimental replicates (e.g., 0 versus 100 μm from the surface). However, conclusions regarding differences in expression of different genes (e.g., *narG* versus *algD*) cannot be drawn due to differences in lasers and detectors used for each gene being analyzed. Results from a replicate experiment are shown in Fig. S5.

Using the agar block biofilm assay (ABBA) (22), we grew mucoid and nonmucoid aggregates suspended in an LB agar medium supplemented with 5 mM nitrate for 16 h, fixed the aggregates in paraformaldehyde, and measured *narG* and *algD* gene expression with HCR (see supplemental methods). As expected, the mucoid strain expressed *algD* more highly than the nonmucoid strain (Fig. 2A and C to E). Spatially, *algD* expression was highest in the zones within the first 200 μm below the air-agar interface (Fig. 2A and C to E). In this same region, rRNA levels were highest and aggregates were largest, suggesting a higher growth rate in this region (Fig. S4). This is consistent with previous studies by Sønderholm et al. (23) and Wessel et al. (24) that found growth rates were higher in P. aeruginosa aggregates growing near air-agar surfaces, correlating with oxygen availability. Interestingly, *narG* was also expressed more highly in the mucoid than in the nonmucoid strain (Fig. 2D) and was expressed more evenly in aggregates at various depths below the agar surface (Fig. 2A to D and S5). Analysis of individual aggregates in the ABBA experiments showed a ring-like pattern of 16S rRNA gene, *algD*, and *narG* expression, correlating with decreasing oxygen availability from the surface to within the aggregate, as would be expected for aggregates of this size (4, 24). Within individual mucoid and nonmucoid aggregates, *algD* expression was detected in cells ∼5 to 15 μm below the aggregate surfaces but was not detected in the innermost cells within ∼10 μm of the aggregate centers (Fig. 2F and G), and this was generalizable to nearly all aggregates expressing *algD*. In contrast, the innermost cells highly expressed *narG*, but cells within ∼0 to 10 μm of the aggregate surface did not express *narG* (Fig. 2F and G). This led us to hypothesize that *algD* was being expressed by cells experiencing hypoxia just below the aggregate surface and not by cells in the innermost, presumably anoxic, regions of the aggregates.

10.1128/mBio.02622-19.4 FIG S4 ABBA nonmucoid and mucoid aggregates grow faster and are larger near the air-agar interface. Quantification of mean aggregate volumes (A) and rRNA HCR signals (B) per nonmucoid PA14 (open bars) and mucoid FRD1 (closed bars) ABBA biofilm aggregate at different depths below the agar surface in each ABBA sample in biological replicates 1 (left) and 2 (right). Each bar represents the mean aggregate volume or HCR signal per aggregate in a 50-μm bin depth below the agar surface, error bars indicate SEMs (*P < 0.0001, unpaired two-tailed *t* test). Download FIG S4, TIF file, 0.5 MB. Copyright © 2019 Jorth et al. 2019 Jorth et al. This content is distributed under the terms of the Creative Commons Attribution 4.0 International license.

10.1128/mBio.02622-19.5 FIG S5 Alginate and nitrate reductase gene expression is generally higher in mucoid P. aeruginosa than in nonmucoid P. aeruginosa in 2 ABBA experiments. Three-dimensional fluorescence micrographs of nonmucoid P. aeruginosa PA14 and mucoid P. aeruginosa FRD1 ABBA samples probed with the Eub338 (rRNA; orange), *algD* (magenta), and *narG* (cyan) HCR v3.0 probes. Shown are biological replicates 1 (A) and 2 (B). Scale bars, 100 μm. Mean *algD* (magenta) and *narG* (cyan) HCR signal per aggregate at different depths below the air-agar interface for nonmucoid PA14 (C and E) and mucoid FRD1 (D and F) in biological replicates 1 (C and D) and 2 (E and F). Quantification of mean *algD* (H and J) and *narG* (G and I) HCR signals per nonmucoid PA14 (open bars) and mucoid FRD1 (closed bars) ABBA biofilm aggregates at different depths below the agar surface in each ABBA sample in biological replicates 1 (G and H) and 2 (I and J). Each bar represents the mean HCR signal per aggregate in a 50-μm bin depth below the agar surface, error bars indicate SEMs (*P < 0.0001, unpaired two-tailed *t* test). Download FIG S5, TIF file, 1.6 MB. Copyright © 2019 Jorth et al. 2019 Jorth et al. This content is distributed under the terms of the Creative Commons Attribution 4.0 International license.

To test where cells were expressing *algD* relative to oxygen availability, we used a microelectrode to measure oxygen concentrations from 0 to 600 μm below the nitrate-supplemented LB agar surface in mucoid and nonmucoid ABBA experiments after 16 h of growth. Unexpectedly, the mucoid strain showed a modest increase in its oxygen consumption rate compared to that of the nonmucoid strain (Fig. 2H). However, as we predicted, the mucoid strain expressed *algD* highest in hypoxic regions (5 to 200 μM oxygen) of the agar, from 0 to 350 μm below the agar surface, and maximally at ∼75 μM oxygen (Fig. 2I and J). In regions with less than 5 μM oxygen, *algD* expression plummeted to <1% of the maximum value detected (Fig. 2I and J). This was surprising because in planktonic cultures, we found that anoxia most strongly induced *algD* expression compared to that under oxic and hypoxic conditions (Fig. S6), similar to previous research (19). Thus, alginate gene expression patterns differ between planktonic and aggregate cells: in aggregate cells, *algD* expression is greatest under hypoxic rather than anoxic conditions.

10.1128/mBio.02622-19.6 FIG S6 Alginate gene expression is induced most highly in planktonic cultures by anoxia. Expression of *algD* was determined for nonmucoid P. aeruginosa PA14 and mucoid P. aeruginosa FRD1 exposed to 0 to 110 μM oxygen after overnight growth under oxic conditions via reverse transcription-quantitative PCR (RT-qPCR). Values are normalized relative to PA14 *algD* expression after exposure to anoxic (0 μM) conditions. Mean values represent triplicate technical replicates from two independent biological replicates, error bars indicates SEMs. Download FIG S6, TIF file, 0.1 MB. Copyright © 2019 Jorth et al. 2019 Jorth et al. This content is distributed under the terms of the Creative Commons Attribution 4.0 International license.

### Conclusions.

These data show that nonmucoid and mucoid P. aeruginosa strains express alginate genes in hypoxic zones, which goes against the general thought that alginate gene expression is constitutive in mucoid P. aeruginosa. Furthermore, these experiments demonstrate the utility of HCR v3.0 for analog quantitation of bacterial gene expression *in situ* at spatial scales relevant to microbial assemblages. Going forward, it will be exciting to combine HCR with tissue clearing methods such as MiPACT (10) to determine whether the expression patterns observed in these *in vitro* studies similarly characterize aggregate populations in more complex environments, such as in the study of pathogens *in vivo*. Direct insight into how pathogen physiology develops in infected tissues, or any other context where spatial observation of microbial activities is important, promises to yield insights that will facilitate more effective control of these communities. Many applications of HCR v3.0 can be envisioned, such as using this visualization tool to analyze microbes after therapeutic interventions to identify bacterial subpopulations that either resist or succumb to treatment; identification of the subpopulations that survive a specific perturbation could be used to guide the development and implementation of future therapeutics. Importantly, the potential utility of HCR 3.0 transcends applications in the realm of pathogenesis and stands to aid the study of microbial activities at the single-cell level in diverse contexts.

### Materials and methods.

Bacterial strains were routinely grown in Luria-Bertani (LB) broth and agar. Bacterial cloning, ABBA experiments, HCR analyses, and oxygen measurements were performed as described previously (10, 13, 22, 25–28). For experimental details see supplemental methods and tables including probe sequences (Table S1), bacterial strains (Table S2), and primers (Table S3).

10.1128/mBio.02622-19.8 TABLE S1 HCR probe target sequences. Download Table S1, DOCX file, 0.1 MB. Copyright © 2019 Jorth et al. 2019 Jorth et al. This content is distributed under the terms of the Creative Commons Attribution 4.0 International license.

10.1128/mBio.02622-19.9 TABLE S2 Bacterial strains. Download Table S2, DOCX file, 0.1 MB. Copyright © 2019 Jorth et al. 2019 Jorth et al. This content is distributed under the terms of the Creative Commons Attribution 4.0 International license.

10.1128/mBio.02622-19.10 TABLE S3 Primers. Download Table S3, DOCX file, 0.1 MB. Copyright © 2019 Jorth et al. 2019 Jorth et al. This content is distributed under the terms of the Creative Commons Attribution 4.0 International license.

10.1128/mBio.02622-19.7 TEXT S1 Supplemental methods. Download Text S1, DOCX file, 0.1 MB. Copyright © 2019 Jorth et al. 2019 Jorth et al. This content is distributed under the terms of the Creative Commons Attribution 4.0 International license.
