# Supplementary figures and images for: Quantitative Visualization of Gene Expression in Mucoid and Nonmucoid Pseudomonas aeruginosa Aggregates Reveals Localized Peak Expression of Alginate in the Hypoxic Zone
Source: mBio. 2019 Dec 17;10(6):e02622-19. doi: 10.1128/mBio.02622-19 (PMC6918079; doi:10.1128/mBio.02622-19)

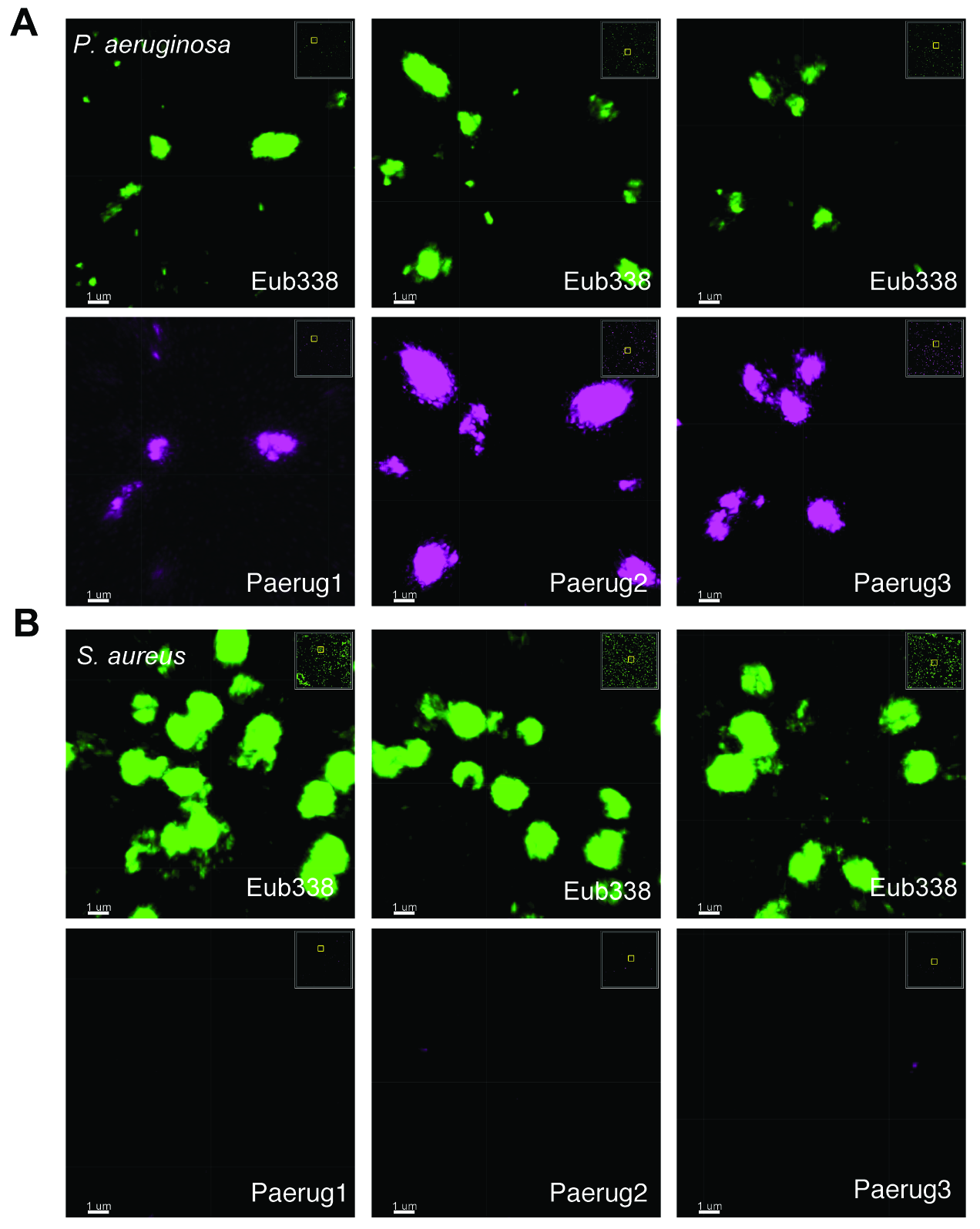

Supplement: FIG S1 [file mBio.02622-19-sf001.tif]

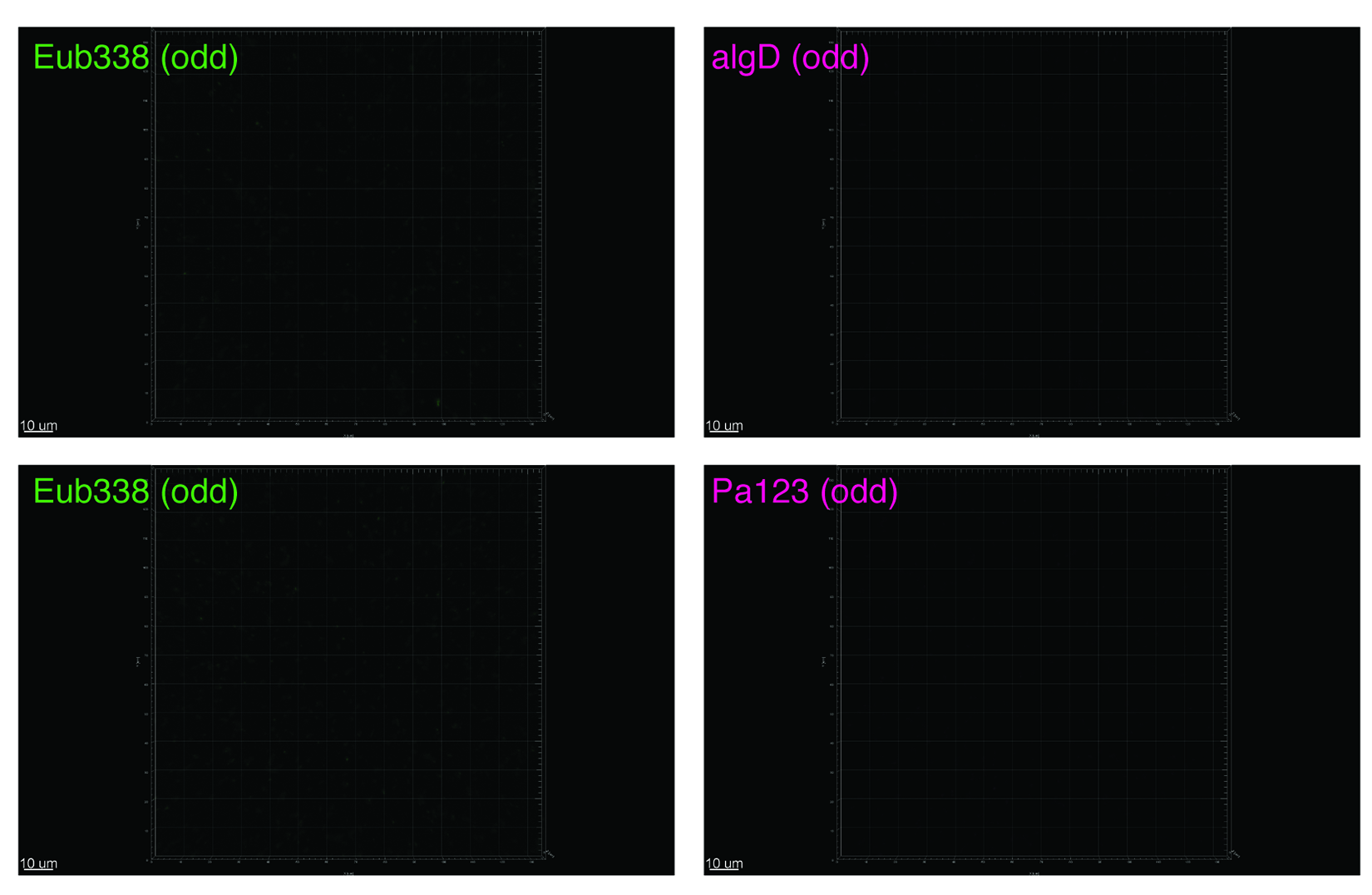

Supplement: FIG S2 [file mBio.02622-19-sf002.tif]

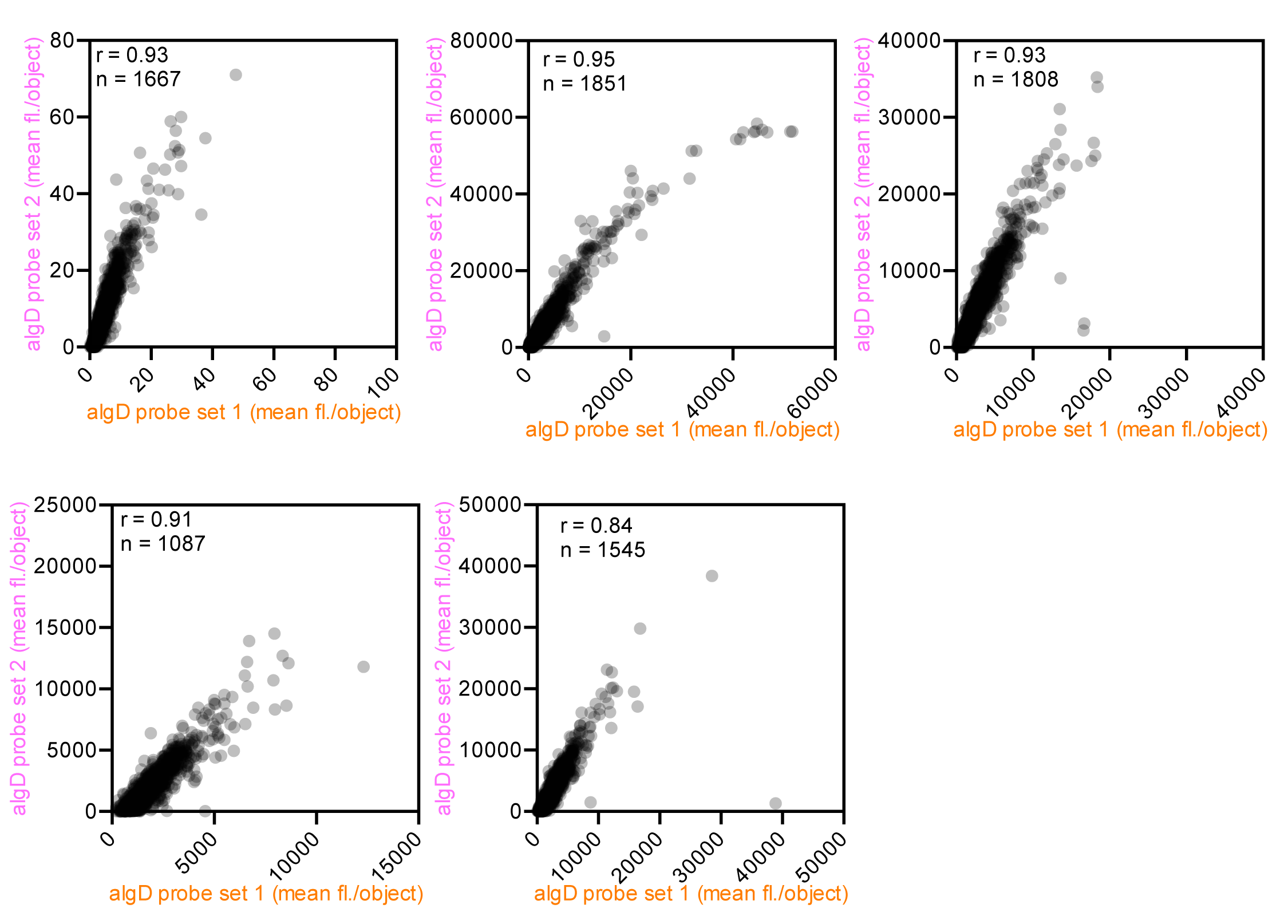

Supplement: FIG S3 [file mBio.02622-19-sf003.tif]

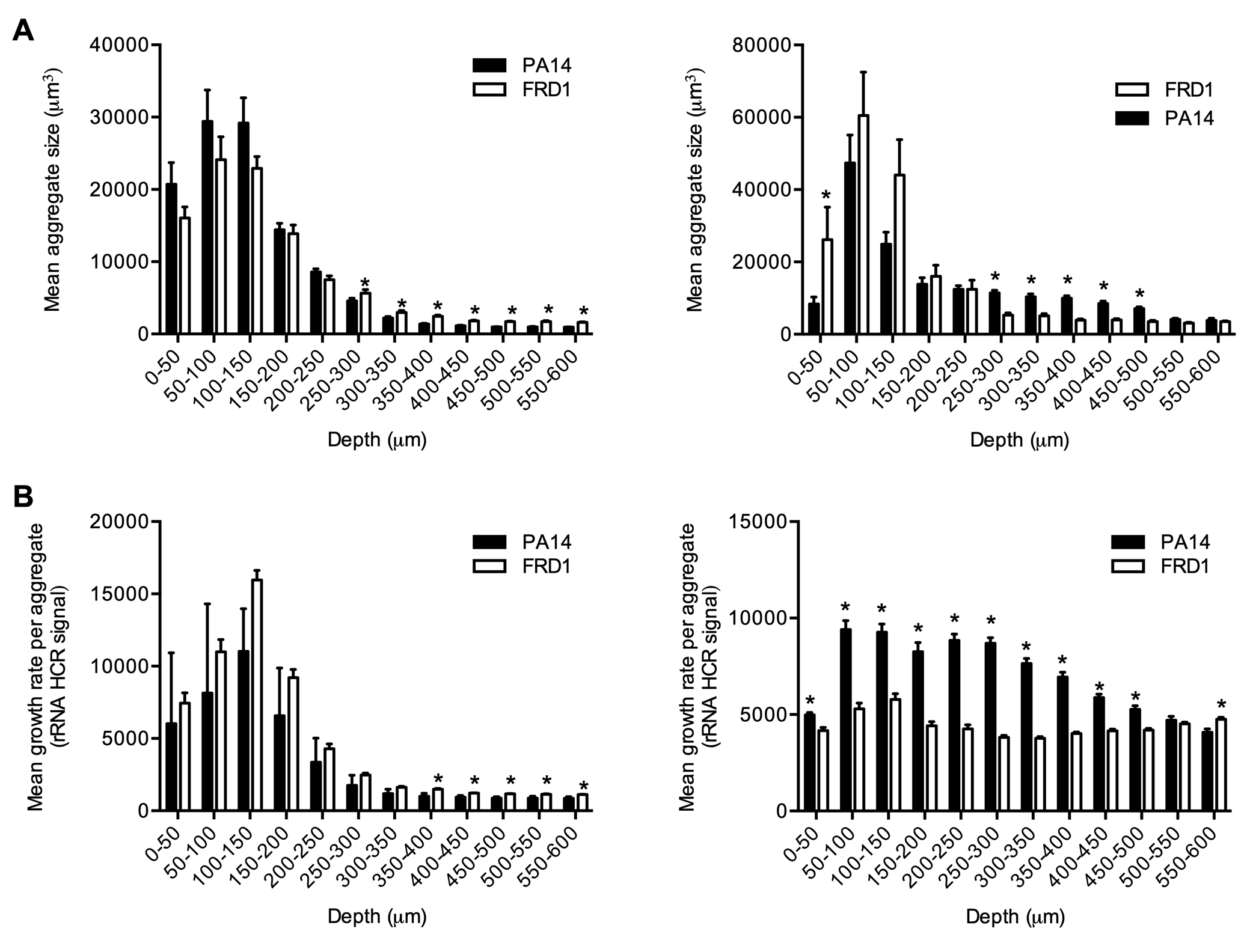

Supplement: FIG S4 [file mBio.02622-19-sf004.tif]

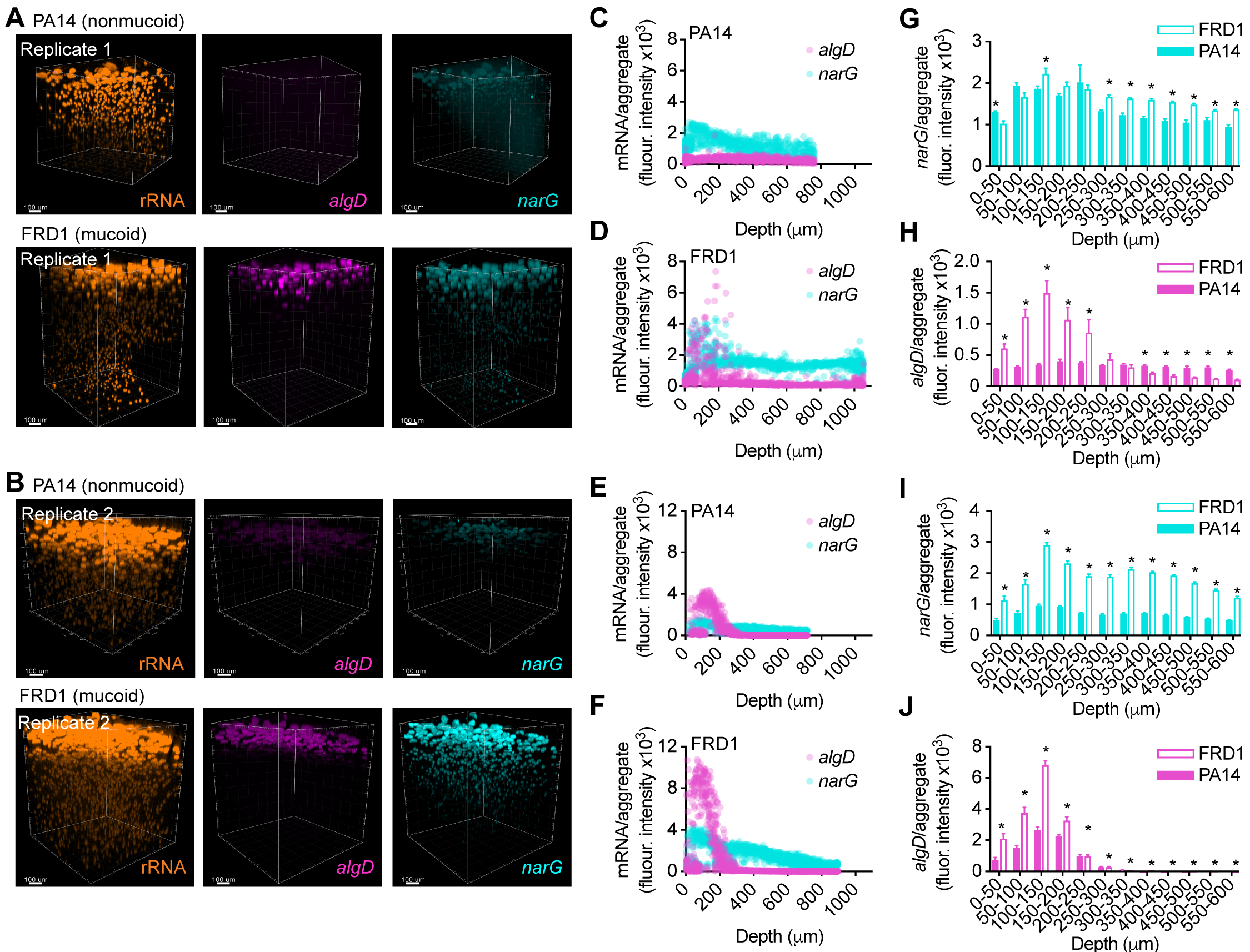

Supplement: FIG S5 [file mBio.02622-19-sf005.tif]

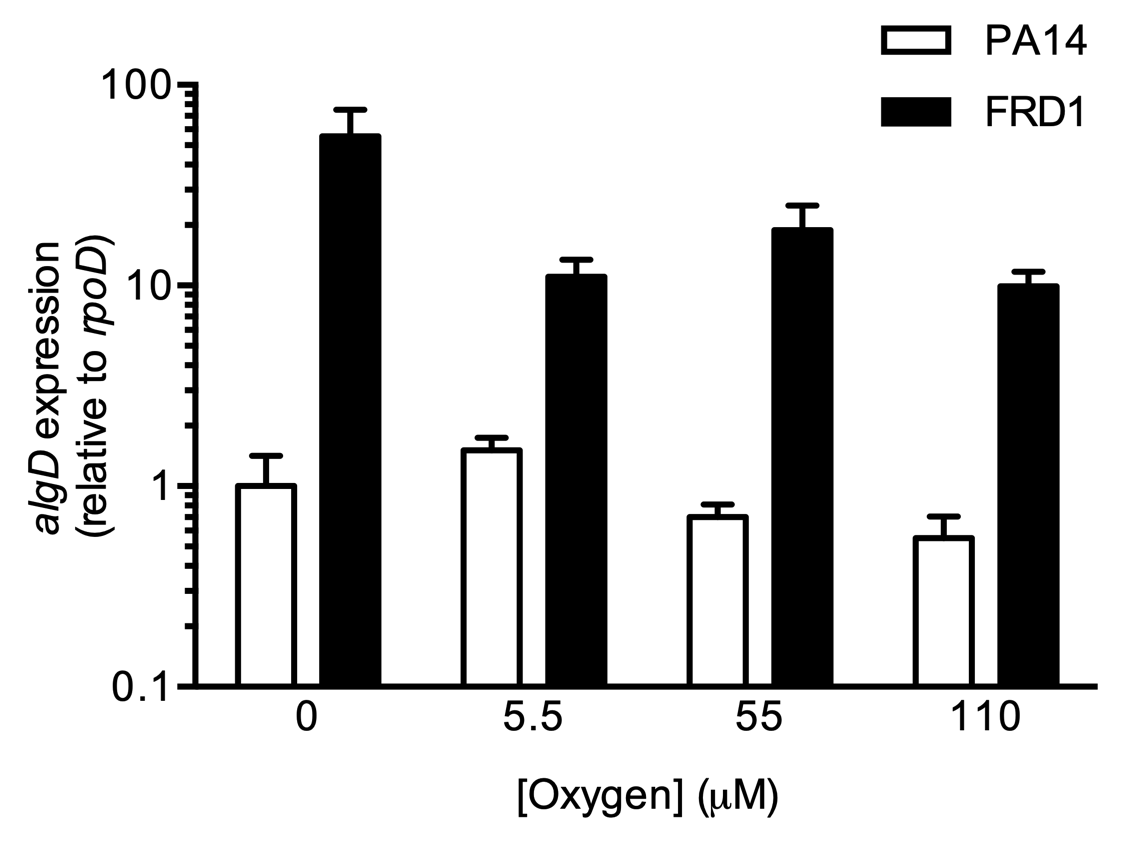

Supplement: FIG S6 [file mBio.02622-19-sf006.tif]
