# Supplementary material for: Quantitative Visualization of Gene Expression in Mucoid and Nonmucoid Pseudomonas aeruginosa Aggregates Reveals Localized Peak Expression of Alginate in the Hypoxic Zone
Source: mBio. 2019 Dec 17;10(6):e02622-19. doi: 10.1128/mBio.02622-19 (PMC6918079; doi:10.1128/mBio.02622-19)
Supplement: TABLE S1 [file mBio.02622-19-st001.docx]

**Table S1. HCR Probe target sequences.**

| **Probe** | **Target sequence** |
| --- | --- |
| Eub338-long | acactggaactgagacacggcccagtcctacgggaggcagcagtggggaa |
| Paerug1 | tcttcggacctcacgctatcagatgagcctaggtcggattagctagttggtg |
| Paerug2 | gccttgacatgctgagaactttccagatggattggtgccttcgggaactc |
| Paerug3 | tagctagtctaaccgcaagggggacttaccacggagtgattcatgactgg |
| narG 1 | atgagtcacctgctcgaccgcctgcagttcttcaagaagaagcagggcgaat |
| narG 2 | cggcagcgctggcagcacgacaagatcgtgcgctccacccacggggtgaact |
| narG 3 | accggctcctgctcctggaagatctacgtgaagaacggcctgatcacctggg |
| narG 4 | ctgaagtacccgaaggtgcgcaagccgttgctcaagctctggcgcgaggcgc |
| narG 5 | tgggacgaggtcaccgagatcatcgccgcggccaacgtctacaccgccaaga |
| narG 6 | gtgcgctacaagggcaccaagaccgtctccatcaccccggactattccgagg |
| narG 7 | ggcatggccttcggtcacgtgatcctgaaggaattccacctcgaccggccga |
| narG 8 | gcctacttcgtcgactactgccgccagtacaccgacatgccgatgctggtgt |
| narG 9 | cagacgcgcctgcaactgtcgctgctcgatggcccggaacatgcctgcgagg |
| narG 10 | gccttcccgtatttcgccgggcaggagcacccgcacttcaagggcgtcgcca |
| narG 11 | atggtgatcatcggcgcggcgatgaaccactggtaccacatggacatgaact |
| narG 12 | agcttcttctacctgcacagctcgcaatggcgccacgagaagctgtcgatgc |
| narG 13 | ccggacgatccgcagaacttcccgcgcaacatgttcatctggcgctccaacc |
| narG 14 | gaggtcgactgggttgacgacggtgccgagggcaagctcgacctggtcacca |
| narG 15 | ctggacttccgcatgtcctccacctgcatgtactcggacatcgtcctgccga |
| narG 16 | gctacctggtacgagaaggacgacctcaacacctccgacatgcaccccttca |
| narG 17 | gtctaccgcaagttcacctcgctcggtccgctgctggacaagctgggcaacg |
| narG 18 | ggcaagggcatcggctggaacaccgagaaggaagtgaagctggtcggcgacc |
| narG 19 | gtcgcggtcaaggcctgggaagcgctgtcgaagatcaccggccgcgagcatg |
| narG 20 | cacctggcgctgcccaaggaagacgagaagatccgcttccgcgacatccagg |
| algD 1 | atgcgaatcagcatctttggtttgggctatgtcggtgcagtatgtgctggct |
| algD 2 | ctgtcggcacgcggtcatgaagtcattggtgtggatgtctccagcaccaaga |
| algD 3 | gacctgatcaaccagggcaagtcgcccatcgtcgaaccgggcctggaagcgt |
| algD 4 | ttgcagcaaggccggcagaccggacggctgtcgggcaccaccgacttcaaga |
| algD 5 | gccgtgctggactccgacgtatcgttcatctgcgtcggcacgccgagcaaga |
| algD 6 | aacggcgacctggacctgggctacatcgagaccgtctgccgcgagatcggct |
| algD 7 | gccatccgcgagaagtccgaacgccacaccgtggtggtgcgcagcaccgtac |
| algD 8 | ccgggcaccgtcaacaacgtggtgatcccgctgatcgaggactgctcgggca |
| algD 9 | aaggccggggtcgacttcggcgtcggcaccaaccccgaattcctccgcgaga |
| algD 10 | accgcgatcaaggactacgacttcccgccgatgaccgtgatcggcgaactgg |
| algD 11 | aagcagaccggcgaccttctcgaggaaatctaccgcgagctggacgcgccga |
| algD 12 | atccgcaagaccgtcgaggtcgccgagatgatcaagtacacctgcaacgtct |
| algD 13 | cacgccgccaaggtcaccttcgccaacgagatcggcaacatcgccaaggcgg |
| algD 14 | ctgtcgcgctactacatgcgtcccggcttcgccttcggcggctcctgcctgc |
| algD 15 | atgctcggttcgttgatgcgcagcaactccaaccaggtgcagaaggccttcg |
| algD 16 | ctcatcaccagccacgacacccgcaaggtcggcctgctcggcctgtcgttca |
| algD 17 | atcggcaagggctacgagttgcgcatcttcgaccgcaacgtcgaatacgcgc |
| algD 18 | gtccacggggccaacaaggaatacatcgagtcgaagatcccgcacgtctcct |
| algD 19 | ctgctggtctccgacctcgacgaagtggtggcgagttccgatgtgctggtgc |
| algD 20 | ggcaatggcgacgagctgttcgtcgacctggtgaacaagaccccgagcggca |
