# Supplementary material for: Quantitative Visualization of Gene Expression in Mucoid and Nonmucoid Pseudomonas aeruginosa Aggregates Reveals Localized Peak Expression of Alginate in the Hypoxic Zone
Source: mBio. 2019 Dec 17;10(6):e02622-19. doi: 10.1128/mBio.02622-19 (PMC6918079; doi:10.1128/mBio.02622-19)
Supplement: TABLE S2 [file mBio.02622-19-st002.docx]

**Table S2. Bacterial strains**

| **Species** | **Strain** | **Source** |
| --- | --- | --- |
| *P. aeruginosa* | UCBPP-PA14 | Marvin Whiteley |
| *P. aeruginosa* | PAO1 Δ*algD* | Matthew Parsek |
| *P. aeruginosa* | PAO1 Δ*algD* pMQ72::*algD* | This study |
| *P. aeruginosa* | PAO1 Δ*algD* pMQ72 | This study |
| *P. aeruginosa* | FRD1 | Matthew Parsek |
| *P. aeruginosa* | FRD875 | Matthew Parsek |
| *S. aureus* | MN8 | Newman lab |
| *P. fluorescens* | WCS365 | George O’Toole |
| *E. coli* | DH10B | Newman lab |
| *E. coli* | DH5α pMQ72 | George O’Toole |
| *E. coli* | Top10 pMQ72::*algD* | This study |
