# Supplementary material for: Quantitative Visualization of Gene Expression in Mucoid and Nonmucoid Pseudomonas aeruginosa Aggregates Reveals Localized Peak Expression of Alginate in the Hypoxic Zone
Source: mBio. 2019 Dec 17;10(6):e02622-19. doi: 10.1128/mBio.02622-19 (PMC6918079; doi:10.1128/mBio.02622-19)
Supplement: TABLE S3 [file mBio.02622-19-st003.docx]

**Table S3. Primers.**

| **Primer** | **Sequence** |
| --- | --- |
| pMQ72-1_rev | CCCGGGTACCGAGCTCGA |
| algD_fwd | attcgagctcggtacccgggAGGTGAATGCGATGCGAATC |
| algD_rev | ctagaggatcCCGCTACCAGCAGATGCC |
| pMQ72-2_fwd | ctggtagcggGATCCTCTAGAGTCGACC |
| pMQ72-2_rev | tagattggtatatatacgcaTATGTAGTGTTGAAGAAACATG |
| pMQ72-1_fwd | TGCGTATATATACCAATCTAAGTC |
| algD-qPCR-F | GGGCTATGTCGGTGCAGTAT |
| algD-qPCR-R | AACGATACGTCGGAGTCCAG |
| rpoD-qPCR-F | CGCAACAGCAATCTCGTCTGAAA |
| rpoD-qPCR-R | GCGGATGATGTCTTCCACCTGTT |
