# Supplementary material for: Quantitative Visualization of Gene Expression in Mucoid and Nonmucoid Pseudomonas aeruginosa Aggregates Reveals Localized Peak Expression of Alginate in the Hypoxic Zone
Source: mBio. 2019 Dec 17;10(6):e02622-19. doi: 10.1128/mBio.02622-19 (PMC6918079; doi:10.1128/mBio.02622-19)
Supplement: TEXT S1 [file mBio.02622-19-s0001.docx]

**Supplemental methods**

***Microbial strains and growth conditions***

*P. aeruginosa, Escherichia coli, P. fluorescens,* and *S. aureus* strains were routinely grown in Luria Bertani (LB) broth shaking at 37°C. *P. aeruginosa* strains containing pMQ72 or pMQ72::*algD* were grown in LB supplemented with 60 μg/ml gentamicin. *E. coli* strains containing pMQ72 or pMQ72::*algD* were grown in LB supplemented with 20 μg/ml gentamicin. For detailed strain information see Table S2.

***HCR v3.0 probe validation***

Species specificity for the *P. aeruginosa* HCR v3.0 Paerug1, Paerug2, and Paerug3 probe pairs (adapted from previous HCR v2.0 probes PSE227, Pae997, and PsaerA, respectively; (1)) were determined by labeling both *P. aeruginosa* PA14, *E. coli* DH10B, *P. fluorescens* WCS365 and *S. aureus* MN8 with the three Paerug probe pairs in a mixture or as individual probe pairs in combination with the universal bacterial rRNA HCR v3.0 probe pair Eub338-long (adapted from previous HCR v2.0 probes Eub338; (1)), which should label all species. HCR v3.0 probes were acquired from Molecular Technologies, and probe target sequences are listed in Table S1. Labeling was performed on slides as described below.

HCR v3.0 probes trigger amplification by hybridization of adjacent odd and even initiator probe pairs. Amplification specificity was determined by incubating odd initiator probes individually without their even initiator probe mates. These analyses were performed with the odd initiator probes for Eub338-long, the algD probe set, and the Paerug1,2,3 probe mixture. Labeling was performed on slides as described below.

***HCR on poly-lysine coated slides***

*P. aeruginosa*, *E. coli*, *P. fluorescens* and *S. aureus* cultures were grown overnight in LB broth. 1 mL of overnight culture was pelleted and cells were fixed by resuspending in 1 mL of 2% paraformaldehyde in phosphate-buffered saline (PBS) pH 7.2, and incubating resuspensions at 25°C for 90 minutes. After fixing, cells were pelleted, washed with PBS, resuspended in 1mL of a 1:1 ethanol:PBS solution, and stored at -20C. Prior to performing HCR, cell suspensions were washed in PBS and treated with 1 mg/ml lysozyme (catalog number L6876; Sigma) in 10 mM Tris-HCl (pH 7.6) for 1 h at 37°C with shaking. *S. aureus* cells were additionally treated with 0.05 mg/ml lysostaphin (catalog number L7386; Sigma) in 10 mM Tris-HCl (pH 7.6) for 1 h at 37°C with shaking. After these treatments, cells were washed with PBS, resuspended in 1mL of a 1:1 ethanol:PBS solution. HCR labeling on slides was performed by spotting 4 μl of fixed cell suspension onto a microscope slide (catalog number: 3039-002; ThermoFisher) and allowing to dry. Probe solutions were prepared, containing 5 nM of appropriate HCR v3.0 initiator odd and even probe pairs in filter-sterilized hybridization buffer (2X SSC (0.3 M NaCl 0.03 M sodium citrate, pH 7.0), containing 20% formamide, 10% dextran sulfate w/v). Probe solutions were added to each dried cell spot, 20 μl per spot. Probes were allowed to hybridize for 3 h in a humidified slide hybridization chamber at 46°C. During the hybridization incubation, fluorescent hairpin probes (Molecular Technologies) were thawed, each denatured in separate tubes in a thermal cycler for 90s at 95°C and allowed to cool for at least 30 m at 25°C in the dark. Denatured hairpins (10 μl of 3 μM stock each) were added to 220 μl filter-sterilized amplification buffer (2X SSC with 10% dextran sulfate). Following the hybridization step, slides were immediately plunged into a vertical staining jar with pre-warmed 42 mM FISH wash buffer (42 mM NaCl, 20mM Tris-HCl [pH 7.6], 5 mM EDTA [pH 7.2], 0.01% SDS) and incubated for 12 m in a 52°C water bath. Following the wash, slides were immediately plunged into a vertical staining jar with ice-cold ~0°C ultra-purified water, then plunged into a vertical staining jar with -20°C ethanol, and slides were allowed to air-dry at 25°C. 20 μl of hairpin buffer mixture was spotted onto each spot of cells, and slides were incubated 1 h in the dark at 25°C. Following the amplification step, slides were immediately plunged into a vertical staining jar with pre-warmed 337.5 mM FISH wash buffer (337.5 mM NaCl, 20mM Tris-HCl [pH 7.6], 5 mM EDTA [pH 7.2], 0.01% SDS) and incubated for 12 m in 48°C water bath. Following the wash, slides were immediately plunged into a vertical staining jar with ice-cold ~0°C ultra-purified water, then plunged into a vertical staining jar with -20°C ethanol, and slides were allowed to air-dry at 25°C in the dark. Each dried cell spot was then covered with 2.5 μl ProLong Gold Antifade Mountant (catalog number: P36930; ThermoFisher), slides were incubated overnight in the dark at 25°C, and imaged using a Nikon Eclipse Ti2 fluorescence microscope. Fluorescent signals were determined using Fiji v2.0.0 software (ImageJ). Using Imaris v9.3 (Bit Plane), individual bacterial cells were defined using the surfaces function based on the Eub338-long rRNA HCR fluorescent signal. When identifying individual cells, the brightfield phase contrast micrographs were used as a guide to set the fluorescent thresholds the determined the cells shapes. The mean fluorescent intensities for the Eub338-long rRNA and Paerug1-3 HCR probes were analyzed from the individual cell defined by the surfaces and plotted for 3 fields of view using Prism v8 (GraphPad Software). A single representative set of micrographs for the two fluorescent channels and a brightfield micrograph are shown for each species.

***Generation of the inducible* algD *strain***

*P. aeruginosa algD* was cloned into the arabinose inducible expression vector pMQ72 using Gibson assembly (2, 3). The *P. aeruginosa algD* gene, and pMQ72 vector were PCR amplified using HiFi HotStart ReadyMix (Kapa). *P. aeruginosa algD* was amplified as one product with primers algD_fwd and algD_rev (Table S3). Due to the size of the plasmid, pMQ72 was amplified as two overlapping products with pMQ72-1_fwd with pMQ72-1_rev and pMQ72-2_fwd with pMQ72-2_rev (Table S3). PCR products were assembled using Gibson Assembly Master Mix (NEB), according the manufacturer’s protocol, to generate pMQ72::*algD*. The Gibson assembly was incubated with DpnI (NEB) using the manufacturer’s protocol to remove residual whole pMQ72 plasmid template that may have carried over from the PCR reactions. Gibson assemblies were transformed into *E. coli* Top10 cells by electroporation, and transformants were selected on LB agar with 20 μg/ml gentamicin. *E. coli* Top10 pMQ72::*algD* strains were grown in LB with 20 μg/ml gentamicin, plasmids were purified using a Monarch Plasmid Miniprep kit (NEB), and plasmid DNA sequences were confirmed by Sanger sequencing. Purified pMQ72 and pMQ72::*algD* were each transformed into *P. aeruginosa* PAO1 Δ*algD* (generated by Tseng et al. (4)) by electroporation as described previously (5), and transformants were selected on Vogel-Bonner Minimal Medium (6) agar supplemented with 60 μg/ml gentamicin.

***Quantification of induced alginate gene expression with HCR***

*P. aeruginosa* PAO1 Δ*algD* pMQ72::*algD* was grown overnight in LB with 60 μg/ml gentamicin and diluted 1:100 in LB with 60 μg/ml gentamicin with 0.10% L-arabinose to induce *algD* expression. When cultures reached mid-exponential phase (OD_600_ 0.60), 1 ml of culture was mixed with 200 μl 20% paraformaldehyde (Electron Microscopy Sciences) to fix cells, and cells were incubated 17h at 4°C on a tube roller. Fixed cells were centrifuged at 19,100*g* and washed 3 times in 1ml PBS to remove paraformaldehyde. After washes, cells were resuspended in PBS to OD_600_ 10.0 (~10^10^ CFUs/ml). A 4 μl drop of the fixed cell suspension was spotted onto a poly-lysine coated slide and allowed to dry. Cells were probed and labeled with the Eub338-long with an Alexa Fluor 488 amplifier to stain rRNA in Channel 1, and two *algD* HCR v3.0 probe sets with Alexa Fluor 594 and Alexa Fluor 647 amplifiers (Paeru Channels 2 and 3, Table S1), as described above. Labeled cells were imaged with a Zeiss LSM880 confocal microscope in the Caltech Biological Imaging Facility. Fluorescent signals for the two *algD* probe sets were determined in 5 fields using the surfaces function in Imaris v9.3 (Bit Plane). A background subtraction was performed with Imaris to remove background fluorescent signals, then the Eub338-long rRNA signals were used as a mask to define the cell shapes. The mean fluorescent intensities for each cell in each channel were plotted and Pearson correlations were determined using Prism v8 (GraphPad Software).

***ABBA-HCR***

ABBA samples were prepared as described previously (7). Briefly, cultures of *P. aeruginosa* PA14 and FRD1 were grown separately overnight, shaking at 37°C. Cultures were diluted to OD_600_ 0.001 in molten LB with 5 mM KNO_3_ and 0.5% noble agar, and 175 μl of the bacterial agar suspension was transferred to chambered cover glass slides (Thermo Fisher Scientific number 155409) and allowed to solidify at 25°C. Once the agar solidified, ABBA samples were transferred to a humidified chamber and incubated for 16 h at 37°C. At 16 h agar blocks were removed from wells in chambered cover glass slides, gently transferred to 4% paraformaldehyde diluted in PBS and incubated 17 h at 4°C to fix cells. Fixed agar blocks were washed gently 2 times with PBS for 30 m per wash. Two biological replicates were performed.

To label *narG* mRNA, *algD* mRNA, and rRNA, agar blocks were incubated 17 h at 46°C with 5 nM of appropriate HCR v3.0 initiator odd and even probe pairs in 500 μl filter-sterilized hybridization buffer. Excess hybridization probes were removed from agar blocks by incubating for 6 h in pre-warmed 42 mM FISH wash buffer in a 52°C water bath. During the hybridization wash, fluorescent hairpin probes (Molecular Technologies) were thawed, each denatured in separate tubes in a thermal cycler for 90s at 95°C and allowed to cool for at least 30 m at 25°C in the dark. Denatured amplification hairpin probes (5 μl each) were added to 110 μl filter-sterilized amplification buffer for each agar block, and blocks were incubated 17 h in the dark at 25°C. Labeled agar blocks were washed by incubating for 3 h in pre-warmed 337.5 mM FISH wash buffer in a 48°C water bath. Following the wash, blocks were submerged in refractive index matching solution (RIMS) with 10 μg/ml DAPI (RIMS/DAPI: 80% HistoDenz (w/v) (Sigma catalog number D2158) in 20 mM phosphate buffer with 0.1% Tween 20, 0.01% sodium azide pH 7.5, 10 μg/ml DAPI). Blocks were incubated 17 h in RIMS/DAPI in the dark, and labeled blocks were imaged using confocal fluorescence microscopy.

***Quantification of aggregate size and gene expression in ABBA***

Bacterial gene expression was quantified using Imaris v9.2.0 (BitPlane). First bacterial aggregates were defined using the surfaces function in Imaris based on the rRNA HCR signal in each Z-stack using the following parameters: [Algorithm]: Enable Region Of Interest = false, Enable Region Growing = false, Enable Tracking = false; [Source Channel]: Source Channel Index = 2, Enable Smooth = true, Surface Grain Size = 1.06 um, Enable Eliminate Background = true, Diameter Of Largest Sphere = 3.97 um; [Threshold]: Enable Automatic Threshold = false, Manual Threshold Value = 250, Active Threshold = true, Enable Automatic Threshold B = true, Manual Threshold Value B = 19340.7, Active Threshold B = false; [Classify Surfaces]: "Volume" above 348 um^3. This analysis generated volumetric data and Z-depth location for each aggregate, as well as fluorescence intensities for each aggregate for the *algD* and *narG* HCR signals. These values were exported and analyzed in Microsoft Excel and Prism v7.

***Oxygen microelectrode measurements***

Oxygen concentrations were measured in the ABBA samples with *P. aeruginosa* PA14 and FRD1 as described previously (7). Briefly, bacteria were grown in ABBA samples at 37°C for 16 hours in a humidified chamber. Oxygen concentrations were measured using a 25‑μm diameter tip Clark-type amperometric electrode using a picoampere amplifier in multimeter (Unisense). A pre-warmed 37°C oxygen-free 0.1 M sodium hydroxide 0.1M sodium ascorbate solution and a pre-warmed 37°C air-saturated 1% salt solution were used to calibrate and set respective zero-points and atmospheric oxygen (199.4 μM O_2_) concentrations. To measure oxygen concentrations at specific depths in the ABBA samples, the sensor was lowered into the ABBA samples using a motorized micromanipulator. Once the surface of the agar was determined by the point at which oxygen concentrations decreased >0.5% below the baseline oxygen concentration, the sensor was lowered using the manipulator in 10 μm steps through the agar up to 600 μm below the surface, measuring oxygen concentrations for 3 s at each step, with 2 s intervals between steps. Oxygen measurements were made from three biological replicates with three technical replicates and processed using the SensorTrace Pro v3.1.3 software.

***qRT-PCR analysis of algD gene expression***

To test the effects of oxygen concentration on *algD* gene expression in planktonic cultures, *P. aeruginosa* PA14 and FRD1 were grown in LB with 5 mM nitrate aerobically shaking at 37°C. Overnight cultures were subcultured into the same medium and grown aerobically shaking at 37°C until cultures reached OD_500_ = 0.1. Cultures were then moved to an anaerobic glove box, transferred to Balch test tubes, and tubes were sealed. The tubes were removed from the glove box and 0, 0.5, 5, and 10% (0, 5.5, 55, and 110 μM) oxygen concentrations were achieved by replacing the anoxic atmosphere in the sealed Balch test tubes with defined volumes of atmospheric oxygen, as described previously (8). Bacteria were exposed to the new oxygen concentrations for 2 h and were immediately mixed with an equal volume RNA later (Life Technologies) to halt transcription. RNA was isolated using a RNeasy Mini Kit (Qiagen), according to the manufacturer’s instructions. Residual DNA was degraded by treating RNA with RQ1 RNase-free DNase (Promega), according to the manufacturer’s instructions. RNA was reverse transcribed to cDNA with random hexamers (Integrated DNA Technologies) using SuperScript III reverse transcriptase (Life Technologies), according to the manufacturer’s instructions. Expression of *algD* was quantified in each cDNA sample using qPCR using the KAPA SYBR FAST qPCR kit (Kapa Biosystems) and a CFX Touch (Biorad) with algD‑qPCR-F and algD‑qPCR-R primers (Table S3). Data were normalized to *rpoD* expression as a reference control gene with rpoD‑qPCR-F and rpoD‑qPCR-R primers (Table S3). Gene expression was quantified using CFX Maestro Software (Biorad) and data were analyzed with Prism software. The experiment was performed in biological duplicate, and technical triplicates were performed for qRT-PCR analyses.

**References**

1. **DePas WH, Starwalt-Lee R, Van Sambeek L, Ravindra Kumar S, Gradinaru V, Newman DK.** 2016. Exposing the three-dimensional biogeography and metabolic states of pathogens in cystic fibrosis sputum via hydrogel embedding, clearing, and rRNA labeling. MBio **7**.

2. **Gibson DG, Young L, Chuang RY, Venter JC, Hutchison CA, 3rd, Smith HO.** 2009. Enzymatic assembly of DNA molecules up to several hundred kilobases. Nat Methods **6:**343-345.

3. **Shanks RM, Caiazza NC, Hinsa SM, Toutain CM, O'Toole GA.** 2006. *Saccharomyces cerevisiae*-based molecular tool kit for manipulation of genes from gram-negative bacteria. Appl Environ Microbiol **72:**5027-5036.

4. **Tseng BS, Zhang W, Harrison JJ, Quach TP, Song JL, Penterman J, Singh PK, Chopp DL, Packman AI, Parsek MR.** 2013. The extracellular matrix protects *Pseudomonas aeruginosa* biofilms by limiting the penetration of tobramycin. Environ Microbiol **15:**2865-2878.

5. **Smith AW, Iglewski BH.** 1989. Transformation of *Pseudomonas aeruginosa* by electroporation. Nucleic Acids Res **17:**10509.

6. **Vogel HJ, Bonner DM.** 1956. Acetylornithinase of *Escherichia coli*: partial purification and some properties. J Biol Chem **218:**97-106.

7. **Spero MA, Newman DK.** 2018. Chlorate specifically targets oxidant-starved, antibiotic-tolerant populations of *Pseudomonas aeruginosa* biofilms. MBio **9**.

8. **Teal TK, Lies DP, Wold BJ, Newman DK.** 2006. Spatiometabolic stratification of *Shewanella oneidensis* biofilms. Appl Environ Microbiol **72:**7324-7330.
